# Cases of Burns Treated by the Application of Flour

**Published:** 1829-04

**Authors:** J. Marshall

**Affiliations:** Surgeon.


### TREATMENT OF BURNS.

### *Cases of Burns treated by the Application of Flour.*

By J. Marshall, Esq. Surgeon.

The minutes of the following case of a severe and extensive burn, with two slighter ones, may probably be deemed worthy of publicity through the medium of the London Medical Journal, with a view to exemplify the practical effects of a simple but highly efficacious remedy. Previously to entering upon a detail of the symptoms, it is deemed expedient to make a few cursory physiological and practical observations on the *modus curandi* of this remedy.

This mild substance is doubtless preeminent to all others hitherto in use, by imparting immediate ease to the inflamed and irritable surface; it rapidly heals by the scabbing process, in uniting with the discharge from the abraded cutis; and almost instantaneously forms a temporary semi-transparent covering, thereby assisting the natural functions in restoring the epidermis. The advantage becomes evident by stopping a profuse discharge, and the tedious progress of ulceration. That remarkable substance, the animal gluten, peculiarly contained in wheat, seems in this instance to assist the rapid regeneration of the scarfskin, and thus protects the cutis and rete mucosum. The surface of the body being wonderfully supplied by the extension of the cutaneous nerves in the form of a soft pulpy membrane, somewhat resembling the expansion of the optic nerve on the retina, readily affords, it is presumed, an explanation of the great violence offered to the system in all cases of extensive burn or scald.

This topical remedy is equally suitable to either of these accidents, and perhaps eventually will be found useful in many other cutaneous affections. It has been recently tried by me in the case of an infant three months old, who laboured under an inflammation attended with ulceration, pouring forth an ichorous discharge: the parts affected were the lips and chin, the right groin, the scrotum, the inside of the right thigh and leg down to the toes. The result was most satisfactory: some parts healed in a few hours, and the whole surface in three or four days. The thickened state of the scrotum, although unavoidably exposed to the frequent irritation of urine, also yielded.

When the flour has formed the artificial covering, the further application becomes comparatively superfluous; which is perceived by its rolling off. This circumstance may be demonstrated by the following example, which equally applies to the manner in which all the other ulcers were healed. The external surface of the nose, from the destruction of the scarfskin, was ulcerated: in the evening it had ceased to discharge, and was apparently healed; the swelling had likewise subsided, and the part assumed its proper size and form: the flour became unnecessary, no longer resting on its surface. This ulcer was particularly regarded, under an impression that the skin of the face, from its peculiar structure, is susceptible of a greater degree of irritability than other parts.

In the lady's case, when the cuticle was completely renovated in some places, though not generally, it imparted a very peculiar feel to the touch, by resembling the dryness and smoothness of parchment: the whole covering of the biceps muscle of the right arm was thus circumstanced. Probably this may be ascribed to the advanced age and previously emaciated state of the patient. This new healed part was of a dark livid purple, which occurred in many other places, accompanied with a similar sensation.

Mr. B. scalded the back of his hand and fingers with steam: he consulted me four days afterwards. The parts were inflamed and swollen, with three blisters going into a state of suppuration. By applying the flour every hour, in less than two days the swelling, inflammation, and ulceration, were completely cured, although the patient had been many years in the habit of indulging freely in ardent spirits. On extending the hand, the back was corrugated, and the cuticle rather stiff and polished.

A boy scalded the left ankle-joint and upper part of the foot. He had applied Goulard's lotion the first two days; and afterwards a dressing, spread on lint, of red precipitate rubbed down with yellow basilicon. Five days after the accident I saw the patient: the part was highly inflamed, and nearly covered with blisters, which had been injudiciously opened, in a state of rapidly spreading ulceration, with a purulent discharge. He could neither use the joint nor bend the toes, being stiff from painful distention. The stimulating dressing was carefully wiped off, where practicable, and the flour substituted. The youth expressed immediate relief. He was directed to apply it every hour during the day, and as often as he awoke in the night, and, wherever the discharge issued through the layer, to apply the flour more assiduously.

Second day.—The patient had passed a good night. Swelling nearly subsided; the surrounding inflammation gone; the ulcers mostly healed: one of them still contained a portion of fluid, and another, near the inner ankle, gave out a discharge of matter. He moved the toes and ankle-joint freely. The change was very remarkable.

Third day.—The fluctuating matter that appeared on the preceding day was wholly absorbed. Patient free from pain. The ulcer near the inner ankle gave off a trifling discharge. On removing from the surface the coat of flour to inspect the character of the granulation, it was found in a most healthy and healing condition. The frightful aspect of a general, ill-conditioned, and irritable ulcer, which threatened mischief on the first view, was effectually removed.

Fourth day.—The surface of the part affected was washed with tepid water, in order to obtain a full view of its state. The whole was healed, except in two small places, not so large as a horsebean, which were in a healthy healing condition. The new skin was of a red hue, and shining.

Mrs. H., a lady in her eighty-fifth year, possessing a good constitution, but greatly emaciated by age, accidentally set fire to her clothes. Her face was much swollen; the hair, eyebrows, and lashes destroyed. It was impossible to recognise her features. The closed and thickened eyelids were opened with difficulty. The other parts injured were the neck, chest, ribs, the back (exceeding half its length), the arms, from the shoulders to the finger ends. The cuticle was raised into numerous blisters, the size of walnuts, on the swollen fingers, palms, and backs of the hands; the epidermis loosely hanging in flakes or tatters on the back, arms, and ribs. This extensive surface, coloured by various hues of red, yellow, and purple, discharged a profuse ichorous and purulent matter. Skin hot; quick, irritable pulse; white tongue, great thirst, and incessant moaning, arising from her sufferings, accompanied by the most afflicting state of mental anxiety.

It is to be regretted that this state of disease was permitted to remain unassisted full twelve hours. On the free application of the flour to the whole surface, the patient ceased to moan, the spirits revived, and she expressed the greatest relief. The flour was applied every hour, but more frequently wherever an oozing of discharge appeared.

In the evening, the skin was cool; pulse steady, eighty-two; the countenance restored to its natural appearance; injured parts looking much better; the discharge generally reduced; bowels had been relieved by an aperient; tongue moist and clean.

On the following morning, (second day,) the patient was cheerful; had slept four hours during the night; had partaken freely of diluents; tongue clean; pulse seventy-eight, skin temperate.

In the evening, no alteration, but the surface more generally healed, and the discharge almost wholly subsided. From so decided an improvement, and the absence of symptomatic fever, hopes were entertained of recovery.

On the morning of the third day, the lady had passed a tranquil night, with intervals of sleep; the tongue had a brown tinge, but moist. Hitherto a febrifuge draught had been taken occasionally; a tonic was now substituted, with Infus. Rosæ et Spiritus Ætheris Nitrosi, and a gentle laxative.

In the evening, the pulse 100; tongue darker brown; skin hot and dry; respiration hurried. Ordered Inf. Rosæ cum Sulph. Quinæ.

The fourth day.—The bowels open. She had passed a restless night, with muttering delirium; subsultus tendinum; pulse 110, skin hot. These symptoms increased towards night.

On the fifth day, the tongue was black and parched; sordes on the lips; great difficulty of deglutition, speechless and convulsed.

She died about five o'clock the following morning.

As the local affection was so happily relieved, and the symptomatic fever for a time suspended, the immediate cause of death must be attributed to the violent shock the system had sustained, together with extreme old age. The case, however, forcibly illustrates the healing effects of flour. The ease with which it is directed by the dredger, and reapplied, without handling or disturbing the parts affected, may suffice to demonstrate, with the foregoing cases and observations, the superiority it possesses over all former dressings. By checking the progress of severe ulceration, it will effectually prevent the frightful scar, the wry neck, contracted limb, and destruction of parts by sloughing.

53, *Jermyn street, St. James's.*

---

